# Supplementary material for: Goals and the Structure of Experience
Source: ArXiv. 2025 Aug 20:arXiv:2508.15013v1. Preprint. [Version 1] (PMC12393231)
Supplement: Supplement 1 [file NIHPP2508.15013v1-supplement-1.pdf]

A Formal setting

A.1 Telic states as goal-equivalent experiences

We assume the setting of a perception-action cycle, or streams of observation-action pairs representing the interaction between the agent and its environment. We denote by $\mathcal{O}$ and $\mathcal{A}$ the set of possible observations and actions, respectively. An experience sequence, or *experience* for short, is a finite se-

quence of observation-action pairs: $h = o_1, a_1, o_2, a_2, ..., o_n, a_n$. For every non-negative integer, $n \geq 0$, we denote by $\mathcal{H}_n \equiv (\mathcal{O} \times \mathcal{A})^n$ the set of all experiences of length n. The collection of all finite experiences is denoted by $\mathcal{H} = \cup_{n=1}^{\infty} \mathcal{H}_n$. In non-deterministic settings, it will be useful to consider distributions over experiences rather than individual experiences themselves and we denote the set of all probability distributions over finite experiences by $\Delta(\mathcal{H})$. Following [130], we define a *goal* as a binary preference relation over experience distributions. For any pair of experience distributions, $A, B \in \Delta(\mathcal{H})$, we write $A \succeq_g B$ to indicate that experience distribution A is weakly preferred by the agent over B (i.e., that A is at least as desirable as B) with respect to goal g. When $A \succeq_g B$ and $B \succeq_g A$ both hold, A and B are equally preferred with respect to g, denoted as $A \sim_g B$. We observe that $\sim_g$ is an equivalence relation, i.e., it satisfies the following properties:

- Reflexivity: $A \sim_g A$ for all $A \in \Delta(\mathcal{H})$.
- Symmetry: $A \sim_g B$ implies $B \sim_g A$ for all $A, B \in \Delta(\mathcal{H})$.
- Transitivity: if $A \sim_g B$ and $B \sim_g C$ then $A \sim_g C$ for all $A, B, C \in \Delta(\mathcal{H})$.

Therefore, every goal induces a partition of $\Delta(\mathcal{H})$ into disjoint sets of equally desirable experience distributions. For goal g, we define the goal-directed, or *telic*, state representation, $\mathcal{S}_g$, as the partition of experience distributions into equivalence classes it induces:

$$\mathcal{S}_g = \Delta(\mathcal{H}) / \sim_g . \tag{1}$$

In other words, each telic state represents a generalization over all equally desirable experience distributions. This definition captures the intuition that agents need not distinguish between experiences that are equivalent (in a statistical sense) with respect to their goal. Furthermore, since different telic states are, by definition, non-equivalent with respect to $\succeq_g$, the goal g also determines whether a transition between any two telic states brings the agent in closer alignment to, or further away from its goal.

A.2 Learning with telic states

How can telic state representations guide goal-directed behavior? To address this question, we start by defining a *policy*, π, as a distribution over actions

given the past experience sequence and current observation:

$$\pi(a_i|o_1, a_1, ..., o_i). \tag{2}$$

Analogously, we define an *environment*, e, as a distribution over observations given the past experience sequence:

$$e(o_i|o_1, a_1, ..., a_{i-1}). \tag{3}$$

The distribution over experience sequences can be factored, using the chain rule, as follows:

$$P_\pi(o_1, a_1, ..., o_n, a_n) = P(o_1, a_1, ..., o_n, a_n|e, \pi) = \prod_{i=1}^{n} e(o_i|o_1, a_1, ..., a_{i-1})\pi(a_i|o_1, a_1, ..., o_i). \tag{4}$$

Typically, the environment is assumed to be fixed, and hence not explicitly parameterized in $P_\pi(h)$ above. Our definition of telic states as goal-induced equivalence classes can now be extended to equivalence between policy-induced experience distributions as follows:

$$\pi_1 \sim_g \pi_2 \iff P_{\pi_1} \sim_g P_{\pi_2}. \tag{5}$$

The question we are interested in can now be stated as follows: how can an agent learn an efficient policy for reaching a desired telic state? In other words, how can the agent's policy be updated to increase its likelihood of generating experiences that belong to a certain desirable telic state, $S_i \in \mathcal{S}_g$? To answer this question, we begin by writing down the empirical distribution of N experience sequences generated by policy π:

$$\hat{P}_\pi(h) = \frac{|\{k : h_k = h\}|}{N}. \tag{6}$$

We would like to estimate the probability that $\hat{P}_\pi(h)$ belongs to telic state S_i, and update π to increase this probability. A fundamental result from large deviation theory, known as Sanov's theorem [131], shows that this probability decays exponentially with a rate of

$$R = \min_{P \in S_i} D_{KL}(P||P_\pi). \tag{7}$$

Since R determines the probability that experiences sampled from P_π belong to telic state S_i, we refer to it as the *telic distance* from π to S_i. Assuming

now the agent's policy can be expressed using some parameterization θ, the following policy gradient method updates π_θ in a way that minimizes the telic distance, i.e., maximizes the likelihood of generating experiences belonging to telic state S_i:

$$\theta_{t+1} = \theta_t - \eta \nabla_\theta D_{KL}(P_i^\star || P_{\pi_\theta}), \tag{8}$$

where $\eta > 0$ is a learning rate parameter and,

$$P_i^\star = arg \min_{P \in S_i} D_{KL}(P || P_\pi), \tag{9}$$

is called *information projection* of P_π onto S_i, i.e., the distribution in S_i which is closest, in the KL sense, to P_π. Equation 8 thus describes a general policy gradient method for learning with telic state representations.

A.3 Illustrative example: the two-armed bandit

To illustrate our proposed learning algorithm, we compute the goal-directed policy gradient for a fully-tractable bandit learning problem and show that, in this simple case, minimizing telic distance yields a commonly reported empirical choice strategy known as probability-matching. We consider a two-armed bandit in which the set of actions is defined as of choosing a left (L) or right (R) lever and the observations are winning (1) or losing (0):

$$\mathcal{A} = \{L, R\}, \ \mathcal{O} = \{1, 0\}. \tag{10}$$

For simplicity we consider a past-independent policy, π_θ, that is parameterized by the probability of choosing action L:

$$\pi_\theta(L) = \theta, \ \pi_\theta(R) = 1 - \theta. \tag{11}$$

The environment e is specified by the probabilities of winning when choosing L or R, denoted p_L and p_R, respectively:

$$e(1|L) = p_L, \ e(0|L) = 1 - p_L; \ e(1|R) = p_R, \ e(0|R) = 1 - p_R. \tag{12}$$

The likelihood that an experience sequence, h, will be generated by the policy induced distribution P_{π_θ} can be expressed as:

$$P_{\pi_\theta}(h) = \theta^{N_L^h}(1-\theta)^{N_R^h} p_L^{N_{L,1}^h}(1-p_L)^{N_L^h - N_{L,1}^h} p_R^{N_{R,1}^h}(1-p_R)^{N_R^h - N_{R,1}^h}, \tag{13}$$

where N_L^h, N_R^h are the number of times the agents selected the L and R actions, respectively, and $N_{L,1}^h, N_{R,1}^h$ are the number of "win" observations following L and R choices, respectively. For simplicity, we assume that the agents goal is to reach a specific number of wins, so that two policies are equivalent if and only if the expected number of wins obtained by following both is equal:

$$\pi_{\theta_1} \sim_g \pi_{\theta_2} \iff \mathbb{E}_{P_{\pi_{\theta_1}}(h)}\left(\sum_i^N \mathbf{1}_{h_i=1}\right) = \mathbb{E}_{P_{\pi_{\theta_2}}(h)}\left(\sum_i^N \mathbf{1}_{h_i=1}\right), \tag{14}$$

where $N = N_L^h + N_R^h$ is the total number of action/observation pairs and $\mathbf{1}_{h_i=1}$ denotes an indicator function which is one if the ith observation in h is 1 and zero otherwise. Thus, for every $j = 1, ..., N$, the telic state S_j is defined simply as the set of all experience distributions with an expected number of wins equal to j:

$$S_j = \{P(h) : \text{ s.t. } \mathbb{E}_{h\sim P}\left(\sum_i^N \mathbf{1}_{h_i=1}\right) = j\}. \tag{15}$$

The telic distance (Eq.7) between a policy π_θ and S_j is given by:

$$D_{KL}(S_j||P_{\pi_\theta}) = \min_{P\in S_j} D_{KL}(P||P_{\pi_\theta}) = \sum_h P_j^\star(h) \log \frac{P_j^\star(h)}{P_{\pi_\theta}(h)}, \tag{16}$$

where $P_j^\star(h)$ is the distribution in S_j closest to $P_\pi(h)$ in the KL sense, as defined in Eq. 9 above. Using Eq. 13 we can compute the telic distance gradient:

$$\nabla_\theta D_{KL}(S_i||P_{\pi_\theta}) = \frac{\sum_h P_j^\star(h) N_R^h}{1-\theta} - \frac{\sum_h P_j^\star(h) N_L^h}{\theta}, \tag{17}$$

so that the optimal policy for reaching j wins, $\pi_{\theta_j^*}$, is given by:

$$\theta_j^* = \mathbb{E}_{h\sim P_j^\star}\left(\frac{N_L^h}{N_L^h + N_R^h}\right). \tag{18}$$

In words, the policy maximizing the likelihood of reaching telic state S_j, is one matching the expected choice probability of $P_j^\star(h)$. Interestingly, a similar "probability-matching" strategy was found in human iterated binary choice behavior [132].

A.4 Closing the loop: telic state conditioned policies

Above we have assumed that the policy depends on the full past experience but this assumption can be relaxed. Within the current framework, we assume that the agent maintains an estimate of the most likely telic state it is currently in and updates it at each time point. Concretely, given a goal g and a past experience sequence at time t, $h_t = o_1, a_1, ..., o_t$, the agent can estimate its current telic state, i.e., the equivalence class of the experience distribution most likely to have generated h_t:

$$\hat{S}_t(h_t) = [\arg \max_{P \in \Delta(\mathcal{H}_t)} P(h_t)]_{\sim_g}. \tag{19}$$

The policy can now be expressed in terms of the estimated telic state:

$$\pi(a_t | \hat{S}_t(h)), \tag{20}$$

so that in choosing actions the agent generalizes over past experiences that are estimated to originate from the same telic state. Since the borders between telic states are determined by the goal, the same experience may be assigned to different telic states under different goals. This clustering of past experience into estimated telic states is lossy: it ignores goal-irrelevant information, and is not necessarily Markovian. Thus, while it may not be optimal in the Bayesian sense, it provides a self contained account of how goals, i.e., preferences over experience distributions, generate intrinsic (telic) state representation, which in turn provide a foundation for action selection and learning.

A.5 Experience features and discrimination sensitivity

A natural way of representing goals, i.e., preferences over experience distributions, is by comparing the likelihood that experiences generated from different distributions will belong to some subset $\Phi_g \subset \mathcal{H}$ representing some desired property of experiences. For example, for the goal of solving a maze, Φ_g might be the set of all experiences, i.e., path trajectories, that reach the exit. Formally, for two experience distributions, A and B, the agent will prefer the one that is more likely to generate experiences belonging to Φ_g:

$$A \succeq_g B : \sum_{h \in \Phi_g} A(h) \geq \sum_{h \in \Phi_g} B(h).$$

The sensitivity parameter, ϵ, effectively determining the maximum difference, in terms of desirable outcome likelihoods, that the agent is willing to ignore in order to reduce representational complexity. In the maze example, experience distribution A would be preferred over B if it is more likely to generate trajectories that reach the exit. Importantly, Eq. A.5 implies that A and B are equivalent only when $\sum_{h\in\Phi_g} A(h)$ and $\sum_{h\in\Phi_g} B(h)$ are precisely equal, which is unlikely in realistic, noisy environments. A more reasonable assumption is that agents can discriminate sampling likelihoods at some finite sensitivity level, $\epsilon > 0$, such that:

$$A \sim_g^{(\epsilon)} B \iff |\sum_{h\in\Phi_g} A(h) - \sum_{h\in\Phi_g} B(h)| \leq \epsilon. \tag{21}$$

In the maze example, this means that two trajectory distributions are considered equivalent if their respective likelihoods of generating exit-reaching trajectories are within ϵ of each other. As we shall see in the following sections, the discrimination sensitivity parameter, ϵ, controls the tradeoff between the granularity of a telic state representation and the policy complexity needed to reach all telic states.

B Telic-controllability and the goal selection problem

In this section, we introduce the notion of *telic-controllability*, a joint property of an agent and a telic state representation, that characterizes whether or not the agent is able to reach all possible telic states using complexity-limited policy update steps. Towards this, we first define an agent's *policy*, π, as a distribution over actions given the past experience sequence and current observation: $\pi(a_i|o_1, a_1, ..., o_i)$. Assuming a fixed environment, the definition of telic states as goal-induced equivalence classes induces corresponding equivalence classes of policy-induced experience distributions as follows:

$$\pi_1 \sim_g \pi_2 \iff P_{\pi_1} \sim_g P_{\pi_2}. \tag{22}$$

As detailed above, this mapping between policies and telic states provides a unified account of goal-directed learning in terms of the statistical distance between policy-induced distributions and desired telic states. To explore this

notion, we introduce a new property – telic-controllability – that plays a central role in the following sections. A representation is called telic-controllable if any state can be reached using a finite number, N, of complexity-limited policy updates, starting from the agent's default policy, π_0, where the complexity of a policy update step is quantified by the Kullback-Leibler (KL) divergence between the post and pre-update step policies. Formally, we have the following:

Definition (telic-controllability)**.** A telic-state representation, $\mathcal{S}_g$, induced by the goal, g, is *telic-controllable* with respect to a default policy, π_0, and a policy complexity capacity, $\delta \geq 0$, if the following holds:

$$\begin{aligned} &\forall S \in \mathcal{S}_g \; \exists \{\pi_t, S_t\}_{t=0}^{N}, N > 0 \text{ s.t. } \forall t < N \\ &\big(S_t = [P_{\pi_t}]_{\sim_g}\big) \wedge \big(D_{KL}(P_{\pi_{t+1}} || P_{\pi_t}) \leq \delta\big) \wedge \big(S_N = S\big), \end{aligned} \tag{23}$$

where $[P_{\pi_t}]_{\sim_g}$ is the goal-induced equivalence class, i.e., telic state, containing P_{π_t}. This definition generalizes the familiar control theoretic notion of controllability in two important ways. First, it applies to telic states, i.e., classes of distributions over action-outcome trajectories, rather than by n-dimensional vectors – the standard control theoretic setting. Second, it takes into account the complexity capacity limitations of the agent, using information theoretic quantifiers to constrain the maximal complexity of policy update steps an agent can take in attempting to reach one telic state from another. As illustrated in the next section, telic-controllability is a desirable property since it means that agents can flexibly adjust to shifting goals using bounded policy complexity resources.

B.1 State representation learning algorithm

A central feature of our approach is the duality it establishes between goals and state representations. In this section, we utilize this duality to develop an algorithm for learning a telic-controllable state representation, or, equivalently, finding a goal that produces such a state representation. The algorithm receives as inputs the agent's current goal, g (represented, e.g., by an ordered set of desired experience features), and default policy, π_0, along with its policy complexity capacity, δ, and the discrimination sensitivity parameter ϵ. Its output consists of a new goal g' such that $\mathcal{S}_{g'}$ is telic controllable with respect to π_0 and δ. The main idea is to split any unreachable telic state, S, i.e., one that cannot be reached from π_0 using policy update steps

with complexity less than δ. State splitting is accomplished by generating a new, intermediate, telic state, S_M, lying between the agent's default policy induced distribution, P_{π_0}, and its information projection on the unreachable telic state, i.e., the distribution $P^* \in S$ that is closest to P_{π_0}, in the KL sense. The intermediate telic state, S_M, is then defined as the set of all distributions that are ϵ-equivalent to P_M (Eq. 21), where P_M is the convex combination of P^* and P_{π_0} lying at a KL distance of δ from P_{π_0}. After generating the new state, S_M, the goal is updated to reflect the proper ordering between the default policy state S_0, the intermediate state S_M, and the originally unreachable state S, such that elements of S_M are between S_0 and S in terms of preference. Pseudocode for the learning algorithm is provided in Algorithm 1. The algorithm makes use of an auxiliary procedure, FindReachableStates (Algorithm 2), to find all reachable states, given the agent's goal, g, default policy, π_0, and policy complexity constraint, δ. This auxiliary procedure performs a recursive search, similar to depth-first search methods, attempting to find policies that are closest, in the KL sense, to currently unreachable telic states, while still sufficiently close to the agent's current policy, as not to exceed the policy complexity capacity. Its main optimization step (line 3) can be implemented, e.g., using policy gradient over the information projection of P_{π_0} on S.

B.2 Illustrative example: dual goal navigation task

In this section, we illustrate the proposed telic state representation framework and learning algorithm outlined above using a simple navigation task in which an agent performs a one dimensional random walk, starting at location $x_0 = 0$, with the goal of reaching one of two non overlapping regions of interest after a fixed number, $T = 30$, of steps. The agent's policy is defined as a stochastic mapping between its current and next position and is parameterized by the mean and standard deviation (μ and σ, respectively) of a Gaussian update step: $\pi(x_{t+1}|x_t; \mu, \sigma) = x_t + \eta_t, \quad \eta_t \sim \mathcal{N}(\mu, \sigma)$. For brevity, we denote by $\pi(\mu, \sigma)$ a policy with a $\mathcal{N}(\mu, \sigma)$ distributed noise term. A graphical illustration of the task and sample trajectories for different policies is shown in Fig. 3.

Since the sum of normally distributed variables is also normally distributed, a policy $\pi(\mu, \sigma)$ induces a Gaussian distribution over the final lo-

Algorithm 1 Telic-controllable state representation learning

Input: π_0: default policy, g: current goal,
δ: policy complexity capacity, ϵ: sensitivity.

Output: g': new goal such that $\mathcal{S}_{g'}$ is telic-controllable with respect to π_0 and δ

$\mathcal{R} \leftarrow [P_{\pi_0}]_{\sim_g}$ ▷ initialize reachable state set
$g' \leftarrow g$ ▷ initialize new goal
while $\mathcal{R} \neq \mathcal{S}_{g'}$ **do**
  $\mathcal{R} \leftarrow \textsc{FindReachableStates}(\pi_0, g', \delta)$ ▷ see algorithm 2 below
  for $S \in \mathcal{S}_{g'} \setminus \mathcal{R}$ **do** ▷ for each unreachable state
    $P^* \leftarrow \arg\min_{P \in S} D_{KL}(P||P_{\pi_0})$ ▷ information projection of P_{π_0} on S
    $M = \arg\max_{t \in [0,1]} t$ s.t. $D_{KL}\big((tP^* + (1-t)P_{\pi_0})||P_{\pi_0}\big) \leq \delta$
    $P_M = MP^* + (1-M)P_{\pi_0}$ ▷ convex combination of P^* and P_{π_0}
    $S_M \leftarrow \{P : P \sim_g^{(\epsilon)} P_M\}$ ▷ ϵ-neighborhood of P_M
    if $P_{\pi_0} \leq_g P^*$ **then** ▷ update goal with preference order for S_M
      $g' \leftarrow g' \cup \{(p,q)_{\leq_{g'}} \in S_M \times S\} \cup \{(r,p)_{\leq_{g'}} \in S_0 \times S_M\}$
    else if $P^* \leq_g P_{\pi_0}$ **then**
      $g' \leftarrow g' \cup \{(q,p)_{\leq_{g'}} \in S \times S_M\} \cup \{(p,r)_{\leq_{g'}} \in S_M \times S_0\}$
    end if
  end for
end while
return g'

cation of the agent:

$$p(x_T \mid x_0 = 0; \mu, \sigma) = \mathcal{N}(T\mu, \sqrt{T}\sigma). \tag{24}$$

To account for goal-directed behavior, we define a right and a left region of interest, R and L, consisting of unit radius segments centered around $x_R = 2$ and $x_L = -2$ respectively. Thus, $R = [R_1, R_2] = [1, 3]$ and $L = [L_1, L_2] = [-3, -1]$. For the purpose of this example, we assume that the agent wants to reach R but avoid L, at time T. For example, for a rodent navigating a narrow corridor, R and L may indicate segments of the corridor where a reward (e.g., food) and a punishment (e.g., air puff) are administered, respectively. We can express the agent's goal in terms of preferences over policies by defining $\Delta P(\mu, \sigma) = p(x_T \in R \mid \mu, \sigma) - p(x_T \in L \mid \mu, \sigma)$ as the difference between the probabilities that the agent will reach regions R and

Algorithm 2 Finding reachable states

Input: π_0: initial policy, g: goal,
δ: policy complexity constraint.

Output: all telic states in $\mathcal{S}_g$ reachable from π_0 by δ-complexity limited policy update steps

procedure RecursiveReach$(\pi, g, \delta, \mathcal{R})$
  for $S \in \mathcal{S}_g \setminus \mathcal{R}$ **do** ▷ for every unreached state S
    $\pi_\theta \leftarrow \arg\min_\theta D_{KL}(S || P_{\pi_\theta})$ s.t. $D_{KL}(P_{\pi_\theta} || P_\pi) \leq \delta$ ▷ optimize policy to reach S
    if $[P_{\pi_\theta}]_{\sim_g} \notin \mathcal{R}$ **then** ▷ if new state reached
      $\mathcal{R} \leftarrow \mathcal{R} \cup [P_\pi]_{\sim_g}$ ▷ add current state to reachable set
      $\mathcal{R} \leftarrow$ RecursiveReach$(\pi_\theta, g, \delta, \mathcal{R})$ ▷ continue from current state
    end if
  end for
  return $\mathcal{R}$
end procedure
procedure FindReachableStates(π_0, g, δ)
  $\mathcal{R}_0 \leftarrow [P_{\pi_0}]_{\sim_g}$ ▷ initialize reachable set
  $\mathcal{R} \leftarrow$ RecursiveReach$(\pi_0, g, \delta, \mathcal{R}_0)$ ▷ try to reach all states recursively
  return $\mathcal{R}$ ▷ return set of reachable states
end procedure

L at time T, with a policy $\pi(\mu, \sigma)$. The agent's goal can now be defined as a preference for policies with higher ΔP values. However, as explained above, due to the agent's finite discrimination resolution, it can only detect whether ΔP is above or below the sensitivity threshold, ϵ. Thus, using Eq. 22, the agent's goal, g, can be expressed by the following preference relation over policies, where we denote, for brevity, $\pi(\mu_i, \sigma_i)$ and $\Delta P(\mu_i, \sigma_i)$ as π_i and ΔP_i, respectively, for $i = 1, 2$:

$$\pi_1 \succeq_g \pi_2 \iff \big(\Delta P_1 \geq \epsilon \geq \Delta P_2\big) \vee \big(\Delta P_1 \geq -\epsilon \geq \Delta P_2\big), \tag{25}$$

where first term on the r.h.s. of Eq. 25 captures the *desirability* of R – the agent prefers policies that have a probability *higher* than ϵ of reaching R over ones that do not; while the second term captures the *undesirability* of L – the agent prefers policies that have a probability *lower* than ϵ to reach L than ones that do not. We recall that telic states can be defined by policies that are similarly preferred, under the agent's discrimination threshold, ϵ, which determines the borders between the resulting telic states. The telic state representation for the goal g defined by Eq. 25, and a threshold parameter of $\epsilon = 0.1$ is visualized in Fig. 4 (top left). Telic state S_R (S_L), is shown as a colored region bounded by a dotted green (red) line, consisting of all policies that are more (less) likely to reach R than L by a probability margin of ϵ or more. Policies that are roughly equally likely to reach R or L, i.e., whose difference in ΔP is smaller than ϵ, constitute an additional "default" telic state, S_0 (teal background), in which the agent is agnostic to which region is it more likely to reach.

$$\begin{aligned} S_R &= \{(\mu, \sigma) | \Delta P(\mu, \sigma) \geq \epsilon\}\,, \\ S_L &= \{(\mu, \sigma) | \Delta P(\mu, \sigma) \leq -\epsilon\}\,, \\ S_0 &= \{(\mu, \sigma) || \Delta P(\mu, \sigma)| \leq \epsilon\}\,. \end{aligned} \tag{26}$$

Using Eqs. 24 and 26 we can express each telic state in closed form, for example S_R can be expressed, using the standard error function, $\operatorname{erf}(x) = 2/\sqrt{\pi}\int_0^x e^{-t^2} dt$, as follows:

$$S_R = \{(\mu, \sigma) \Big| \frac{1}{2}\left(\operatorname{erf}\frac{R_1 - T\mu}{\sqrt{2T}\sigma} - \operatorname{erf}\frac{R_2 - T\mu}{\sqrt{2T}\sigma}\right) - \frac{1}{2}\left(\operatorname{erf}\frac{L_1 - T\mu}{\sqrt{2T}\sigma} - \operatorname{erf}\frac{L_2 - T\mu}{\sqrt{2T}\sigma}\right) \geq \epsilon\},$$

with similar expressions for S_L and S_0. To illustrate the notion of telic-controllability (Eq. 23) using this representation, we define the complexity, $C(\pi)$, of a policy, $\pi(\mu, \sigma)$, with respect to the agent's default policy, $\pi_0(\mu_0, \sigma_0)$, as the KL divergence, per time step, between them:

$$C(\pi) \equiv D_{KL}(\pi \| \pi_0).$$

The contour lines in the first three panels of Fig. 4 (top & bottom left) show isometric policy complexity levels for an agent with a complexity capacity of $\delta = 1$ bit per time step, and a default policy $\pi_0(\mu_0 = 0, \sigma_0 = 1)$. Initially, both telic states, S_R, and S_L, lie within the range of the agent's policy complexity capacity (top left). The policies in S_R and S_L that are closest in the KL sense to π_0 (green and red dots, respectively), both lie within a range of less than δ from π_0, i.e., the state representation is telic-controllable. When the center of R shifts from $x_R = 2$ to $x_R = 2.5$ (top right), telic state S_R is no longer within complexity range δ from π_0 and the state representation becomes non-controllable. To address this (bottom left), the state representation learning algorithm described in B.1, splits S_R by adding an intermediate telic state S_M (orange), centered around the policy closest to S_R that is still within a KL-range of δ from π_0 (yellow dot). This changes the shape of S_R and S_L since now the probability of reaching each of the three telic states, S_R, S_L and S_M, is defined in with respect to the two others, e.g., $S_M = \{(\mu, \sigma) | \Delta P_M(\mu, \sigma) \geq \epsilon\}$ where $\Delta P_M = p(x_T \in M \mid \mu, \sigma) - \max\{p(x_T \in L \mid \mu, \sigma), p(x_T \in R \mid \mu, \sigma)\}$, and similarly for S_R and S_L. Since π_M is, by construction, within a KL range of δ from π_0, the agent can reach S_M by updating its default policy to π_M (bottom right), bringing S_R into reach again. Hence, the new state representation, consisting of S_0, S_L, S_M and S_R, is tellic-controllable. Fig. 6 illustrates the telic-complexity curves, showing the probability of reaching each telic state achievable for a given complexity capacity level (x-axis). These curves quantify the maximal gain in the probability of reaching each telic state, S_R, S_M or S_L, relative to the other two (ordinate), for a given policy complexity capacity level, with respect to a default policy of π_0 (left) or π_M (right) (abscissa). Finally, Fig. 5 illustrates the granularity-complexity tradeoff: the granularity of the state representation, quantified as $-\log(\epsilon)$ (abscissa), controls the complexity capacity required to reach each state (ordinate). Finer-grained representations are generally more controllable. For a granularity level of $\epsilon = 0.1$ (gray vertical line), only S_L and S_M are reachable from $\pi_0(0, 1)$ under a complexity capacity of $\delta = 1$ (gray horizontal line).

This work was supported by grant no. U01DA050647 from the National Institute on Drug Abuse and ZIAMH002983 from the Intramural Research Program of the National Institute of Mental Health.

We wish to thank the reviewers for their thoughtful comments and Yael Niv for her feedback and support.
